# Unusual Location of a Cutaneous Granular Cell Tumor: A Case Report and Review of the Literature

**DOI:** 10.7759/cureus.106226

**Published:** 2026-03-31

**Authors:** Maha El Maati, Nawfal Fejjal, Cédric Lenormand, Mouna Rimani, Laila Benzekri

**Affiliations:** 1 Dermatology, Ibn Sina University Hospital, Mohammed V University, Rabat, MAR; 2 Pediatric Plastic Surgery, Ibn Sina University Hospital, Mohammed V University, Rabat, MAR; 3 Dermatology, University Hospital of Strasbourg, Strasbourg, FRA; 4 Pathology, Hassan Pathology Center, Rabat, MAR

**Keywords:** abrikossoff’s tumor, dermatofibrosarcoma protuberans, granular cell tumor, immunohistochemistry, submammary neoplasm

## Abstract

Granular cell tumor (GCT), or Abrikossoff’s tumor, is an uncommon neurogenic neoplasm originating from Schwann cells. While predominantly benign and frequently found in the head and neck, its clinical and radiological presentation can mimic more aggressive soft tissue sarcomas. We report the case of a 65-year-old female patient presenting with a firm, slow-growing mass in the right submammary fold. The biopsy revealed polygonal cells with granular, eosinophilic cytoplasm. Immunohistochemistry (IHC) showed diffuse S100 protein positivity and CD34 negativity, confirming a cutaneous GCT. The tumor exhibited infiltrative growth patterns with involved margins, perineural tracking and vascular invasion that defined an atypical tumor according to Fanburg-Smith criteria. A wide excision with 1 cm margins was made to achieve a complete resection with uneventful follow up. GCT is often known as a “great mimicker” due to its ability to simulate malignancies. Given the high local recurrence rate (20-40% for margin-positive or infiltrative lesions), GCTs necessitate wide-margin excision.

## Introduction

Granular cell tumor (GCT), first described by Alexei Ivanovich Abrikossoff in 1926, is an uncommon neoplasm of soft tissue [1]. Historically known as "myoblastoma," its true origin is now recognized as neurogenic, from Schwann cells. GCTs are predominantly benign; however, criteria have been established for atypical and malignant GCTs. While they occur predominantly in the head and neck, with a predilection for the tongue, a substantial number arise in the skin and subcutaneous tissues [2]. 

We present a case of a cutaneous GCT in the inframammary fold with atypical histological features treated by an enlarged surgical excision. The case underscores the critical role of immunohistochemistry (IHC) and the need for complete surgical excision when aggressive histological features are present [3,4].

## Case presentation

A 65-year-old female patient, with a medical history of insulin-dependent diabetes mellitus, substituted hypothyroidism, and hypercholesterolemia on statins, presented with a mass in her right submammary area. The lesion had been evolving for two years, demonstrating slow, progressive growth with intermittent inflammatory episodes.

**Figure 1:**
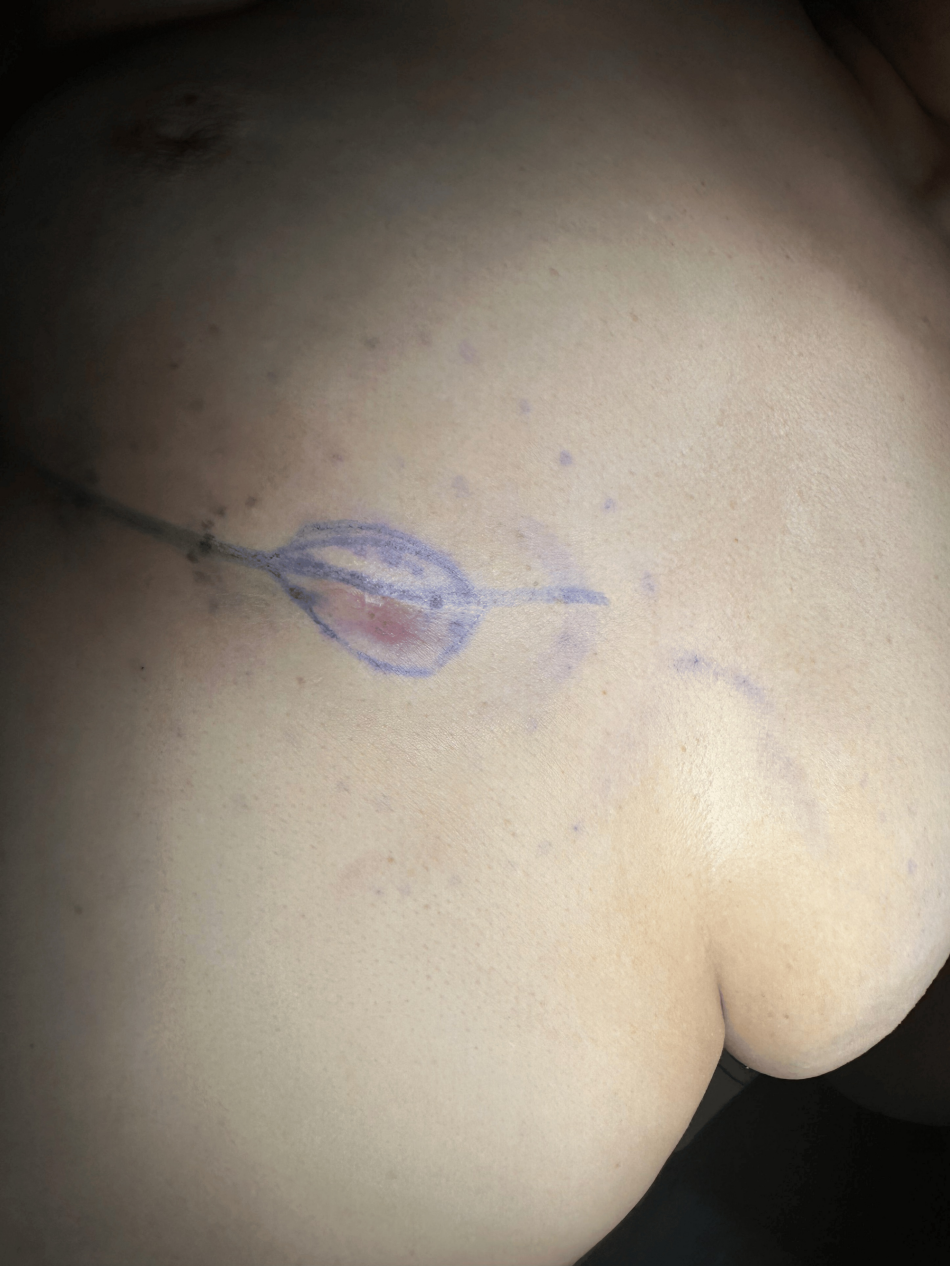
Erythematous nodule on the right inframammary fold surrounded by an induration of subcutaneous tissue

Clinically, it could be described as a firm tumor of 4 cm surrounded by an induration of soft tissues (Figure 1). The ultrasound revealed a cutaneo-subcutaneous thickening. A follow-up magnetic resonance imaging (MRI) characterized an intradermal lesion with a fibrous signal pattern suggestive of a subcutaneous soft tissue sarcoma. 

**Figure 2:**
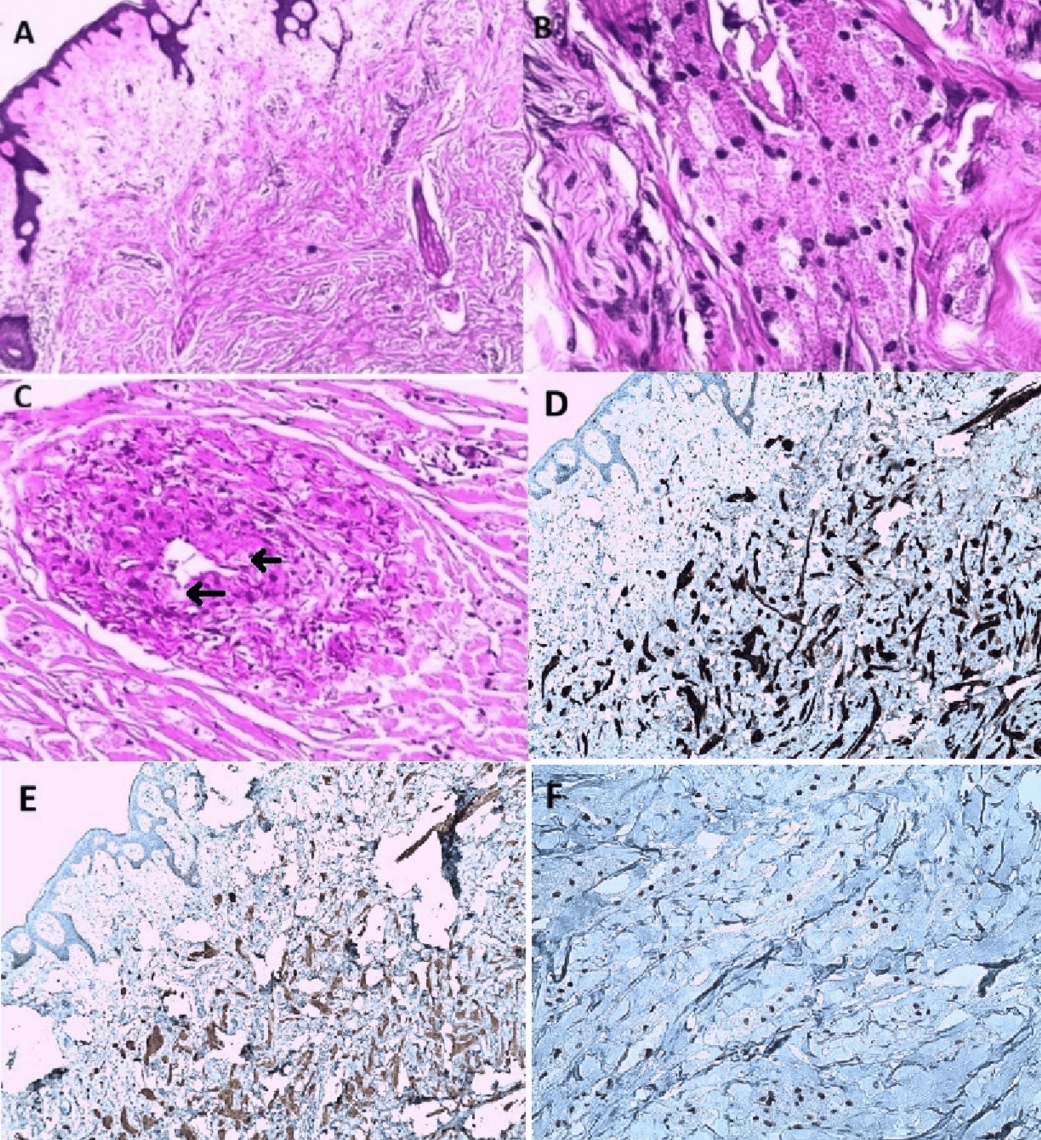
Histological images (A) Image of a histological section after HES staining at 50x magnification showing dermohypodermic proliferation by rounded or oval cells in layers, with large, finely granular eosinophilic cytoplasm and small round central nuclei, developing on a fibrous background. (B) A magnification of 400x shows perineural sheathing. (C) A magnification of x 200 shows subendothelial angioinfiltration (arrow). (D) Positive immunostaining for S100. (E) Positive staining for CD68. (F) Positive nuclear staining for TFE3. HES: haematoxylin-eosin-saffron; TFE3: transcription factor E3

The histological study showed a poorly defined tumor proliferation involving the entire thickness of the reticular dermis and extending into the underlying hypodermis. It was composed of sheets of oval cells with abundant, granular, eosinophilic cytoplasm. Within the lesion, images of perineural sheath involvement, vascular invasion, and involvement of some arrector pili muscles were observed. The cells developed against a background of dermal fibrosis, which contained a few deep perivascular lymphocytic infiltrates. The inferior and deep surgical margins were focally involved (Figure 2).

An additional IHC study was performed showing a diffuse expression of the S100 protein CD68 and neuron-specific enolase (NSE) and a nuclear diffuse expression of TFE3. The anti-melanoma antibody HMB45 was not expressed. The mitotic index Ki67 was expressed in 2% of tumor cells. The final histopathology report was consistent with an atypical cutaneous poorly circumscribed GCT with evidence of perineural tracking and vascular invasion (Figure 2).

Following the multidisciplinary tumor board consultation, a second-stage wide excision with 1 cm margins was recommended and subsequently performed to ensure complete clearance. The patient had an uneventful follow-up after eight months.

## Discussion

GCTs are a rare, usually benign, slow-growing neoplasm derived from Schwann cells. It typically presents as a solitary, firm, painless nodule in the head and neck region (especially the tongue). It can occur in the subcutaneous region presenting as an elastic well-defined or ill-defined mass, with a thickening and dimpling of the skin above. The thoracic region can also be affected with breast pain and fixation to the pectoralis muscle [5].

Clinically and radiologically, a GCT in the breast region can simulate numerous neoplasms (as detailed in Table 1). In the sub-mammary fold area, the extreme thickening of the epidermis above a GCT can be mistaken for squamous cell carcinoma (SCC). The granular cells beneath and S100 clarify the diagnosis [6].

**Table 1:** Differential diagnosis of cutaneous GCT with key distinguishing features Table Credit: Maha El Maati References: [7,8] GCT: granular cell tumor; PEH: pseudoepitheliomatous hyperplasia; PAS: periodic acid–Schiff; NSA: neuron-specific enolase; PAS: periodic acid–Schiff; GCDFP-15: gross cystic disease fluid protein 15

Differential Diagnoses	Key Morphological Features	Immunohistochemical (IHC) Profile
Granular Cell Tumor	Large polygonal cells with abundant granular eosinophilic cytoplasm. Small, central, round nuclei. Pustulo-ovoid bodies of Milian (large PAS+ inclusions). Frequent PEH of the overlying epidermis.	S100 (diffuse/strong +), SOX10 (+), CD68 (+) (due to lysosomes), NSE (+), Calretinin (+/-), PAS/D (+). Negative for: cytokeratins, desmin.
Granular/Apocrine Breast Carcinoma	More infiltrative growth pattern. Cytoplasm is granular and eosinophilic with a higher nuclear-to-cytoplasmic ratio, prominent nucleoli, and nuclear pleomorphism.	Pan-cytokeratins (AE1/AE3) (+), GATA3 (+), androgen receptor (+), GCDFP-15 (+), Mammaglobin (+). Negative for: S100.
Granular Cell Dermatofibroma	Spindle cell proliferation at the periphery with "collagen trapping." Granular changes are often focal. Overlying epidermal induction (not just PEH)	CD163 (+), Factor XIIIa (+/-), CD68 (+). negative for: S100, SOX10
Amelanotic Melanoma (Granular variant)	Significant cytologic atypia, high mitotic rate, and necrotic areas. May show a junctional component or nested architecture.	S100 (+), SOX10 (+), Melan-A (+), HMB-45 (+), MiTF (+). Note: S100 alone does not differentiate GCT from melanoma.
Granular Cell Leiomyoma	Intersecting fascicles of spindle cells with "cigar-shaped" nuclei and granular cytoplasm.	Desmin (+), Smooth Muscle Actin (SMA) (+), h-Caldesmon (+). Negative for: S100.

Most GCTs are benign. However, Fanburg-Smith et al. defined six histologic criteria: spindling, necrosis, high nuclear to cytoplasmic (N:C) ratio, vesicular nuclei with large nucleoli, increased mitotic activity, and pleomorphism [7]. Neoplasms that met three or more of these criteria were classified as histologically malignant; those that met one or two criteria were classified as atypical; and those that displayed only focal pleomorphism but fulfilled none of the other criteria were classified as benign [7,8].

The critical step in our case was the IHC demonstration of strong S100, CD68, and positivity (Schwannian origin) and CD34 negativity, which definitively ruled out differential diagnoses.

The treatment for GCTs is complete surgical excision. The management strategy is heavily dictated by the status of the margins. In fact, GCTs are notoriously ill-defined and lack encapsulation. Even benign GCTs have a local recurrence rate that can reach 20-40% if the margins are involved [9]. Our case, despite being pathologically benign, displayed perineural tracking and vascular invasion with involved margins denoting a locally infiltrative behavior and necessitating cautious management.

## Conclusions

The clinical and radiological presentation of a GCT in the inframammary fold often mirrors more aggressive malignancies, such as breast carcinoma. This case highlights the important role of IHC in achieving a definitive diagnosis and ruling out morphologically similar mimics. Furthermore, while most of GCTs are benign, the presence of infiltrative features such as perineural tracking and vascular invasion, as seen in our patient, warrants a more cautious approach. These findings, even in the absence of full malignancy criteria, underscore the locally aggressive potential of atypical GCTs. Therefore, a multidisciplinary approach and a commitment to achieving wide, clear surgical margins are essential to minimize the significant risk of local recurrence and ensure favorable long-term outcomes.
